# Dataset on hydrophobicity indices and differential scanning calorimetry thermograms for poly(HEMA)-based hydrogels

**DOI:** 10.1016/j.dib.2019.103891

**Published:** 2019-04-28

**Authors:** Ankita Bhat, Blake Smith, Cerasela-Zoica Dinu, Anthony Guiseppi-Elie

**Affiliations:** a Center for Bioelectronics, Biosensors and Biochips (C3B^®^), Department of Biomedical Engineering, Texas A&M University, College Station, TX 77843, USA; b Department of Biomedical Engineering, Texas A&M University, College Station, TX 77843, USA; c Department of Chemical Engineering, West Virginia University, Morgantown, WV 26506, USA; d ABTECH Scientific, Inc., Biotechnology Research Park, 800 East Leigh Street, Richmond, VA 23219, USA

## Abstract

Hydrophobicity indices for poly(HEMA)-based hydrogels: HEMA, AEMA, and DMAEMA calculated from two different methods: 1) Partition coefficients, and 2) Kyte-Doolittle scale are depicted.

Thermograms from differential scanning calorimetry of poly(HEMA)-based hydrogels containing AEMA, DMAEMA, and a mixture of AEMA and DMAEMA are included to represent the glass transition temperature (T_g_) values of the hydrogels.

More information on the methodology to calculate the hydrophobicity indices using the aforementioned methods and the procedure for using a differential scanning calorimeter and analysis of a thermogram is described.

Details of how the changes in the feed composition of poly(HEMA)-based hydrogels was made is provided in the research article ‘MOLECULAR ENGINEERING OF POLY(HEMA-co-PEGMA)-BASED HYDROGELS: ROLE OF MINOR AEMA AND DMAEMA INCLUSION’ (Bhat et al., 2019).[1].

**Table:** Specifications table

Subject area	*Biomedical Engineering*
More specific subject area	*Responsive biomaterials*
Type of data	*Tables, images (thermograms)*
How data was acquired	*Differential scanning calorimeter (Q2000, TA Instruments, New Castle, DE) with accompanying TA Universal Analysis software.*
Data format	*Analyzed*
Experimental factors	4 mol*% HEMA,* 4 mol*% AEMA,* 4 mol*% DMAEMA, and* 2 mol*% AEMA +* 2 mol*% DMAEMA were analyzed using differential scanning calorimetry (DSC) with an associated software. To determine the glass transition temperature (T*_*g*_*) of the hydrogels, samples were first dehydrated.*
Experimental features	*To determine the glass transition temperature (T*_*g*_*) of the hydrogels, samples were first dehydrated then placed and sealed into hermetic pans (Tzero hermetic lid, 901684.901; Tzero pan, 901683.901), equilibrated at -20°C and heated to 200°C at* 10 °C/min *for two cycles. The first cycle was performed in order to erase the thermal history of the hydrogels, and the second cycle was performed in order to determine the inherent thermal properties of the hydrogels. The T*_*g*_*was determined by extrapolation of thermal trace data using TA Universal Analysis software.*
Data source location	Center for Bioelectronics, Biosensors and Biochips (C3B^®^)*, Department of Biomedical Engineering, Texas A&M University, College Station, Texas, United States of America.*
Data accessibility	*Data is with this article.*
Related research article	*A. Bhat, B. Smith, C.-Z. Dinu, A. Guiseppi-Elie, Molecular engineering of poly (HEMA-co-PEGMA)-based hydrogels: Role of minor AEMA and DMAEMA inclusion, Materials Science and Engineering: C, 98 (2019) 89–100.*

**Table:** 

**Value of the data** • The protocol provided for the preparation of poly(HEMA)-based hydrogels, can be compared to other methods of preparation by various researchers. • The hydrophobicity indices for the poly(HEMA)-based hydrogels can be used and cited by other researchers in their fields. • The data provide insights into the glass transition temperatures (Tg) of the poly(HEMA)- based hydrogels, which can be of value to researchers in related fields. • These data can be compared to the glass transition temperatures (Tg) for other types of hydrogels.

## 1 Data

**Fig. 1:**
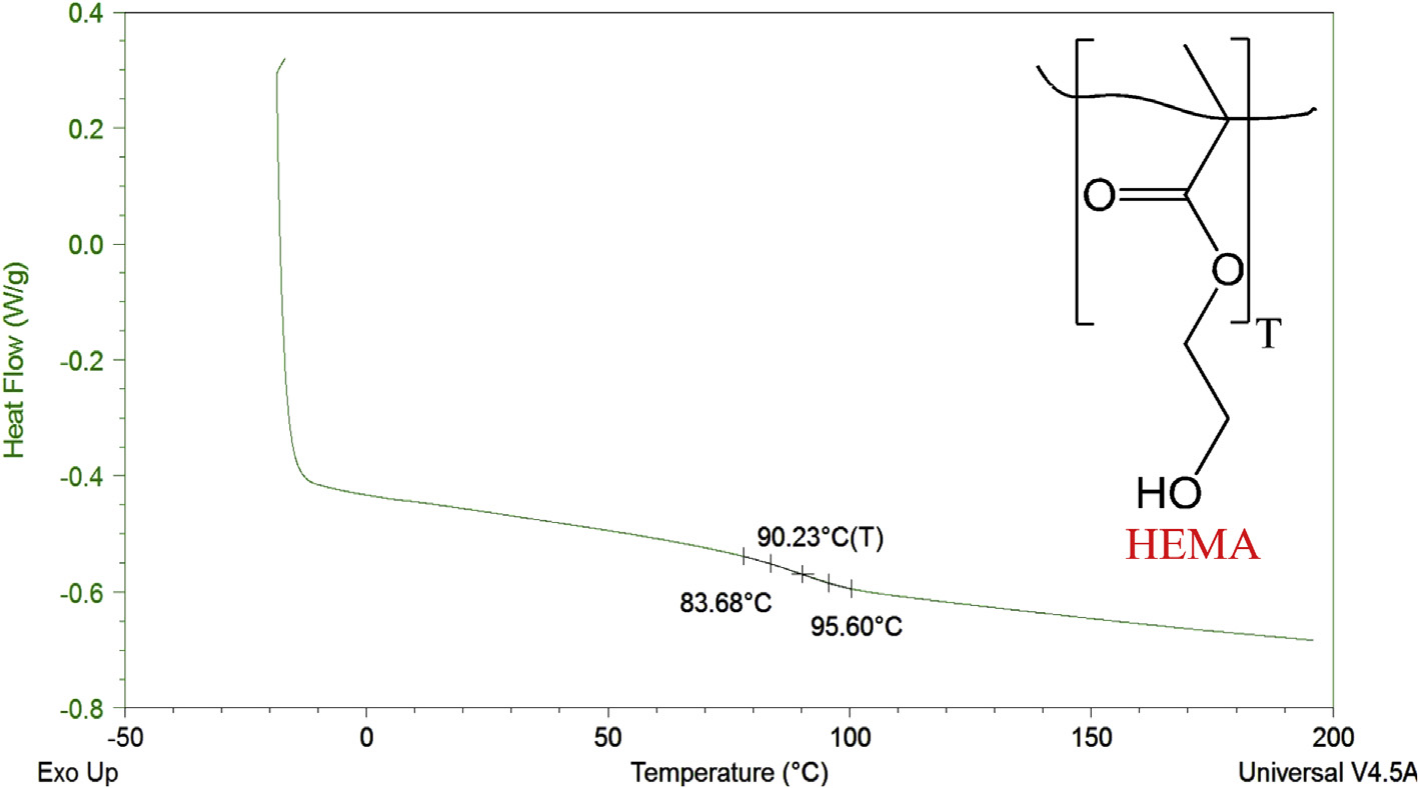
DSC thermogram for poly(HEMA)-based hydrogel containing 4 mol% HEMA.

**Fig. 2:**
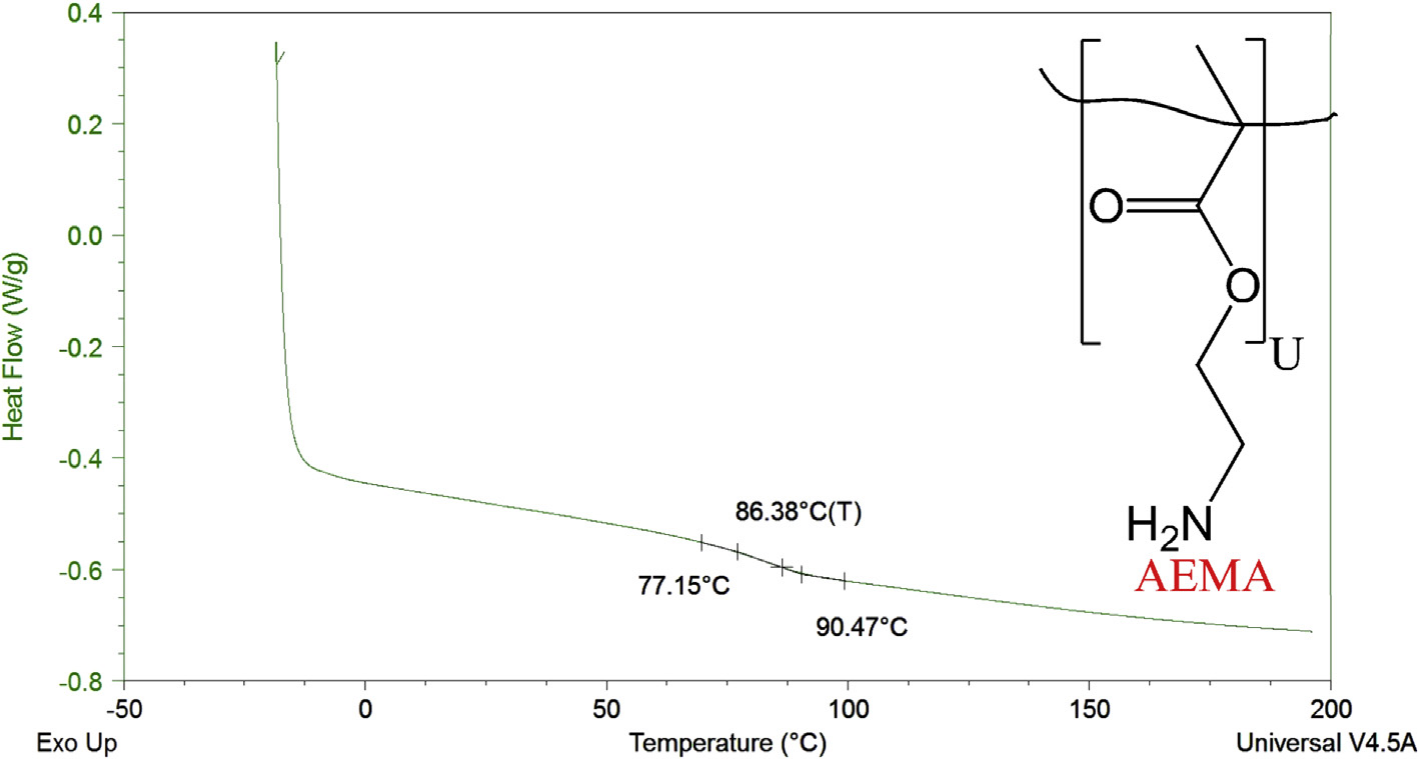
DSC thermogram for poly(HEMA)-based hydrogel containing 4 mol% AEMA.

**Fig. 3:**
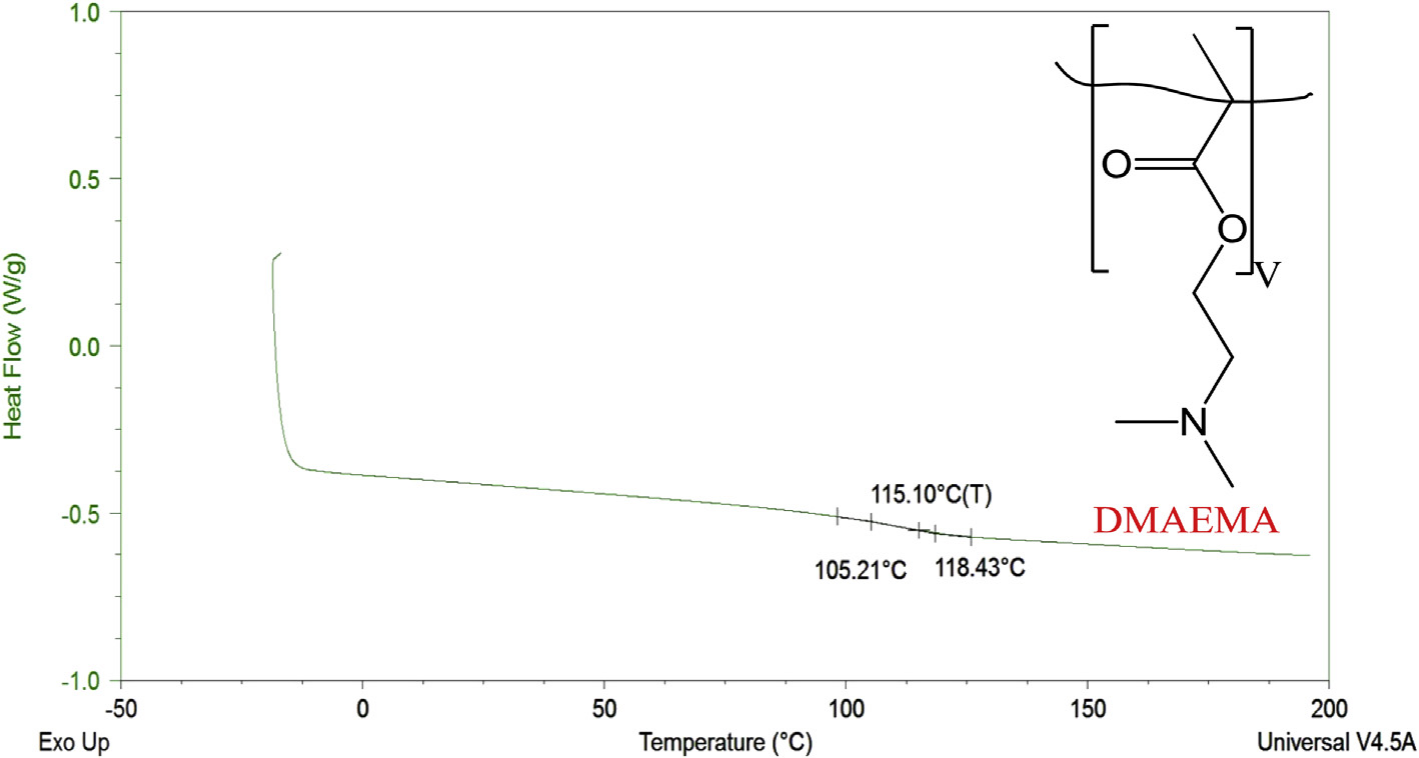
DSC thermogram for poly(HEMA)-based hydrogel containing 4 mol% DMAEMA.

**Fig. 4:**
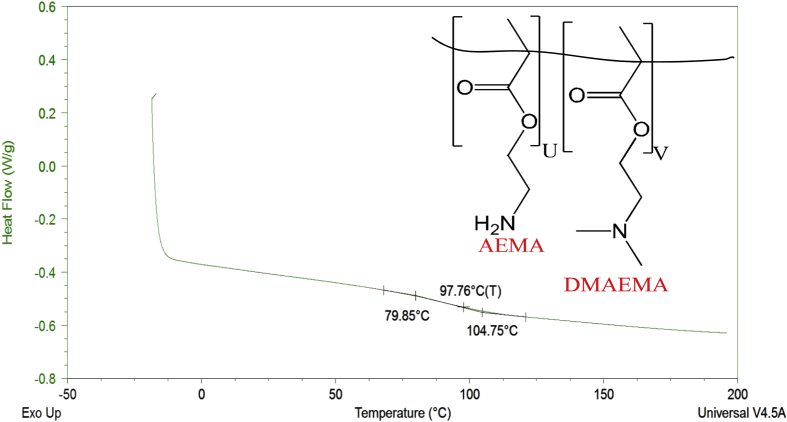
DSC thermogram for poly(HEMA)-based hydrogel containing 2 mol% AEMA+ 2 mol% DMAEMA.

Hydrophobicity indices and differential scanning calorimetry thermograms are described for HEMA, AEMA, and DMAEMA poly(HEMA)-based hydrogels. Hydrophobicity indices are established by two methods. The first method mentions the hydrophobicity indices for the monomers based on the partition coefficients of monomers [2] derived from their functional group contributions. Table 1 lists the hydrophobicity indices using the first method. The second method determines the hydrophobicity indices for the monomers based on comparisons of their functional groups with the Kyte-Doolittle scale [3] for amino acids. Table 2 shows the hydrophobicity indices using the second method. Fig. 1, Fig. 2, Fig. 3, Fig. 4. Depict the differential scanning calorimetry thermograms for poly(HEMA)-based hydrogel polymers synthesized to contain 4 mol% HEMA, 4 mol% AEMA, 4 mol% DMAEMA, and 2 mol% AEMA plus 2 mol% DMAEMA. Table 3 shows the glass transition temperature, T_g_, for all four poly(HEMA)-based hydrogel formulations.

**Table 1:** Partition coefficients of monomers based on their functional group contributions.

Monomers	Functional group	Partition coefficients (log P)
HEMA (CH_3_OH)	—OH	−0.74
AEMA (CH_3_NH_2_)	—NH_2_	−0.57
DMAEMA (N(CH_3_)_3_	-N(CH_3_)_2_	0.16

**Table 2:** Determining hydrophobicity indices of monomers as per comparison of functional groups with Kyte-Doolittle scale for amino acids.

Monomers	Functional group	Partition coefficient (log P)	Amino acid	Hydrophobicity index
HEMA	—OH	−0.74	Ser	−0.8
AEMA	—NH_2_	−0.57	Asn and Lys	−3.5 and -3.9
DMAEMA	-N(CH_3_)_2_	0.16	Leu and Arg	3.8 and -4.5

**Table 3:** Glass transition temperature, T_g_, for all four poly(HEMA)-based hydrogel formulations containing 4 mol% HEMA, 4 mol% AEMA, 4 mol% DMAEMA, and 2 mol% AEMA + 2 mol% DMAEMA (n = 3, mean ± 95% C.I.) [1].

Property	4 mol% HEMA	4 mol% AEMA	4 mol% DMAEMA	2 mol% AEMA 2 mol% DMAEMA
T_g_(°C)	93.2 ± 2.9	86.3 ± 1.3	114.2 ± 0.7	96.3 ± 0.4

## 2 Experimental design, materials, and methods

### 2.1 Preparation and synthesis for poly(HEMA)-based hydrogels

The monomers 2-hydroxyethyl methacrylate (HEMA), poly(ethylene glycol)(360)methacrylate (PEG(360)MA), N-[tris(hydroxymethyl)methyl]acrylamide (HMMA, 93%), N-(2-aminoethyl) methacrylamide (AEMA, 90%), N,N-(2-dimethylamino)ethyl methacrylamide (DMAEMA, 98%), the cross-linker tetra(ethylene glycol) diacrylate (TEGDA, technical grade), the biocompatible viscosity modifier polyvinylpyrrolidone (pNVP, MW ∼1,300,000) and the photo-initiator 2,2- dimethoxy-2-phenylacetophenone (DMPA, 99+%) were purchased from Sigma Aldrich Co. (St. Louis, MO, USA). Methacrylate and diacrylate reagents were passed through an activated alumna inhibitor removal column (306312, Sigma-Aldrich Co., St. Louis, MO) in order to remove the polymerization inhibitors hydroquinone and monomethyl ether hydroquinone. The buffer formed from 4-(2-hydroxyethyl)-1-piperazineethanesulfonic acid sodium salt (HEPES) was prepared to physiologically relevant conditions of 25 mM and pH = 7.4. A Milli-Q^®^ plus (Millipore Inc., Bedford, MA) ultrapure water system was used to prepare deionized water. All other common chemicals and solvents were purchased from Sigma Aldrich Co. (St. Louis, MO, USA) and were used as received, unless otherwise stated.

Hydrogel constituents and their exact molar composition are as tabulated in Table 4 were handled in a UV-free laboratory with UV filtering sleeves (TG-T8TG-UV, Lightbulbsurplus.com) placed over the fluorescent light bulbs. Four unique hydrogel pre-polymer formulations were prepared that varied in composition and were synthesized from HEMA, AEMA and DMAEMA by varying 4 mol% (nominally) of the responsive and cationogenic constituent. Thus, all hydrogels comprised 80 mol% HEMA. The formulation referenced as 4 mol% HEMA contained an additional 4 mol% HEMA to a total of 84 mol% HEMA and served as a reference formulation. Other hydrogels were formulated by replacing the 4 mol% HEMA with 4 mol% AEMA, 4 mol% DMAEMA, or a mixture of comprising 2 mol% AEMA and 2 mol% DMAEMA. To improve component solubility, a mixed solvent comprising 1:1 (v/v) ratio of ethylene glycol and DI water was added to the mixture such that it comprised 20 vol% of the formulation. Finally, the mixture was ultrasonicated for 5 min and sparged with nitrogen gas to remove dissolved oxygen prior to casting and crosslinking [1].

**Table 4:** Monomer composition (mol%) for all four poly(HEMA)-based hydrogel formulations containing 4 mol% HEMA, 4 mol% AEMA, 4 mol% DMAEMA, and 2 mol% AEMA + 2 mol% DMAEMA [1].

Polymer constituents	Mol% of monomer components			
	4 mol% HEMA	4 mol% AEMA	4 mol% DMAEMA	2 mol% AEMA 2 mol% DMAEMA
HEMA (Base monomer: hydrophilic)	79.8	79.8	79.8	79.8
TEGDA (Cross-linker)	3.3	3.3	3.3	3.3
PEGMA(360) (Confers biocompatibility)	5.8	5.8	5.8	5.8
HMMA (Support monomer: hydrophilic)	4.4	4.4	4.4	4.4
pNVP (Pre-polymer: Increases viscosity) (on the basis of repeat unit structure)	1.9	1.9	1.9	1.9
HEMA (Monomer: hydrophilic)	4.4	0	0	0
AEMA (Monomer: hydrophilic)	0	4.4	0	2.2
DMAEMA (Monomer: hydrophobic)	0	0	4.4	2.2
DMPA (Photoinitiator)	0.4	0.4	0.4	0.4

To prepare hydrogel samples for characterization and testing, the hydrogel formulations were cast inside press-to-seal silicone isolator chambers (JTR12R-2.0, Grace Biolabs, Bend, OR) comprising 12 each of 4.5 mm diameter x 1.6 mm depth that were placed between two hydrophobically prepared glass slides. Prior to casting, both sides of the glass slides were thoroughly degreased with acetone, UV cleaned for 10 min (UV-ozone Cleaner, Boekel Industries Inc., Feasterville, PA) and sonicated in isopropyl alcohol to further remove contaminants. The slides were then plasma cleaned (Plasma cleaner/sterilizer PDC-32 G, Harrick Plasma, Ithaca.

NY) to activate —OH groups and immediately incubated in a freshly prepared solution of 0.1% octadecyltrichlorosilane (OTS) in toluene for 45 minutes. The glass slides were then sonicated in isopropyl alcohol for 5 minutes and the silanol condensation with —OH groups of the glass allowed to proceed in an oven by sequentially heating to 40, 110, and 40 °C for 20 minutes at each temperature. Once cooled to RT the isolator was pressed to one glass slide and each chamber filled with the hydrogel cocktail. A second glass slide was then gently lowered onto the chambers. Hydrogels were UV cross-linked for 5 min (CX-2000, UVP, Upland, CA). Upon completion of cross-linking, the polymerized hydrogels were removed from the glass slides and gradually hydrated and unreacted monomer extracted by soaking for 1 h each in ethanol (99%) and 25 mM HEPES buffer (pH 7.4) mixtures in proportions of 100/0, 75/25, 50/50, 25/75 and 0/100 (mL/mL %) [1].

### 2.2 a) Calculations for partition coefficients

Partition coefficient (unit less), P = [X]_organic_/[X]_aqueous_, is the ratio of molar concentrations (mol/L) in contacting phases [4], generally an organic phase vs the aqueous phase (page 1112, Sangster) [2]. When log P > 0, P > 1; [X]_org_ > [X]_aq_ and when log P < 0, P < 1; [X]_org_ < [X]_aq._

As monomers HEMA, AEMA and DMAEMA differ only with respect to their functional groups OH, NH_2_, and N(CH_3_)_3_ respectively, it is assumed that only these functional groups contribute to the partition coefficients.

For CH_3_OH, CH_3_NH_2_, N(CH_3_)_3_; log P values were calculated to be −0.74 (table 9, page 1150, Sangster), −0.57 (table 15, page 1192, Sangster), 0.16 (table 15, page 1193, Sangster) respectively [2]. For CH_3_OH and CH_3_NH_2_ the log P values are negative indicating hydrophilicity. For N(CH_3_)_3_, log P is 0.16 (positive), indicating hydrophobicity. These values serve to provide a relative ranking of the monomer along a continuum from highly hydrophilic to hydrophobic.

b) Evaluating the hydrophobicity indices of the monomers using Kyte-Doolittle scale (for amino acids) [3].

Kyte-Doolittle scale [3] lists the hydrophobicity indices of amino acids. We allocated these hydrophobicity indices to our monomers based on their functional group similarity with the R-groups of amino acids.

### 2.3 Differential scanning calorimetry (DSC) thermograms

To determine the glass transition temperature (T_g_) of the hydrogels, samples were first dehydrated then placed and sealed into hermetic pans (Tzero hermetic lid, 901684.901; Tzero pan, 901683.901), equilibrated at −20 °C and heated to 200 °C at 10 °C/min for two cycles. The first cycle was performed in order to erase the thermal history of the hydrogels, and the second cycle was performed in order to determine the inherent thermal properties of the hydrogels. The T_g_ was determined by extrapolation of thermal trace data using TA Universal Analysis software.

## References

[1] Bhat A., Smith B., Dinu C.-Z., Guiseppi-Elie A. (2019). Molecular engineering of poly (HEMA-co-PEGMA)- based hydrogels: role of minor AEMA and DMAEMA inclusion. Mater. Sci. Eng. C.

[2] Sangster J. (1989). Octanol-water partition coefficients of simple organic compounds. J. Phys. Chem. Ref. Data.

[3] Kyte J., Doolittle R.F. (1982). A simple method for displaying the hydropathic character of a protein. J. Mol. Biol..

[4] Kotanen C.N., Janagam D.R., Idziak R., Rhym L., Sullivan R., Wilson A.M., Lowe T.L., Guiseppi-Elie A. (2015). Partitioning of coomassie brilliant blue into DMAEMA containing poly(HEMA)-based hydrogels. Eur. Polym. J..

