# Supplementary material for: Dataset on hydrophobicity indices and differential scanning calorimetry thermograms for poly(HEMA)-based hydrogels
Source: Data Brief. 2019 Apr 28;24:103891. doi: 10.1016/j.dib.2019.103891 (PMC6517576; doi:10.1016/j.dib.2019.103891)
Supplement: Multimedia component 1 [file mmc1.docx]

Friday, March 8, 2019

Hao-Ran Wang, Ganhui Lan

Editors-in-Chief,

*Data in Brief*

*MOLECULAR ENGINEERING OF POLY(HEMA)-BASED HYDROGELS: ROLE OF MINOR AEMA AND DMAEMA INCLUSION - SUPPLEMENTAL INFORMATION*

Dear Profs. Wang and Lan:

Conflict of interest form will be submitted on line for *Data in Brief*.


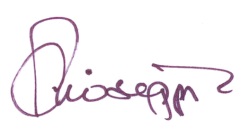
Sincerely,

Prof. Dr. Anthony Guiseppi-Elie, Sc.D., FAIMBE, FRSC, FIEEE

TEES Research Professor of Engineering

Professor, Department of Biomedical Engineering

Professor, Department of Electrical and Computer Engineering
